# Skin manifestations of primary COVID-19 infection with the omicron variant

**DOI:** 10.1371/journal.pone.0352201

**Published:** 2026-07-17

**Authors:** Chuan Ma, Jiachen Sun, Zilian Liu, Chunlei Zhang, Yuansheng Chen, Jiawang Ma, Qing Ma

**Affiliations:** 1 Department of Dermatology, Peking University Third Hospital, Beijing, China; 2 Chinese Center for Disease Control and Prevention, Beijing, China; 3 University at Buffalo, Buffalo, New York, United States of America; Mayo Clinic College of Medicine and Science, UNITED STATES OF AMERICA

## Abstract

**Background:**

Although cutaneous manifestations of COVID-19 have been increasingly documented across earlier variants, the dermatological characteristics specifically associated with the Omicron variant, particularly in the Chinese population, remain insufficiently characterized in the literature.

**Objective:**

To characterize cutaneous manifestations in patients with primary COVID-19 infection caused by the Omicron variant and corresponding management.

**Methods:**

94 participants were included in this retrospective analysis, who developed dermatologic symptoms within one month of COVID-19 Omicron Variant infection.

**Results:**

Mean age was 38 years old, ranging from 7 to 89; with 66% aged within 25–50. A significantly higher incidence of skin manifestations was reported among females (males vs. females, 1:2.76, p = 0.001). Skin rashes typically appeared three days after the onset of COVID symptoms (ranging between 2–7 days), which were mostly mild to moderate. Common morphological types included wheals, erythema, and papular/papulovesicular eruptions. Generalized rashes occurred in 50% of cases, while localized rashes mainly affected the extremities. Pruritus was the most frequent symptom (>90%), particularly in patients presenting with wheals. Clinical management included topical steroid creams and antiviral treatment for COVID-19. No follow-ups were required for most participants (>95%).

**Conclusion:**

Skin rashes associated with Omicron variant COVID-19 infection, predominantly affecting young and middle-aged women, are characterized by wheals, erythema, and papular/papulovesicular eruptions. Early identification and management of these cutaneous symptoms should be integrated in the overall treatment plan of Omicron variant COVID-19 infection.

## Introduction

COVID-19 disease has become a global pandemic since its emergence in late 2019, with various clinical features ranging from asymptomatic to severe acute respiratory syndrome (SARS) [1]. Current evidence has demonstrated that COVID-19 induces a cytokine storm marked by excessive release of pro-inflammatory cytokines [2], which can lead to multisystem organ dysfunction [3]. In the pre-Omicron era, several distinct cutaneous patterns were identified, including: maculopapular eruptions, often described as morbilliform or measles-like; wheel lesions; chilblain-like acral lesions, commonly referred as COVID toes; vesicular eruptions; livedo reticularis-like patterns; and petechial and purpuric rashes [4, 5]. Most of these dermatological disorders, except the chilblain-like lesions, were often temporally associated with the course of the infection. The pathogenesis was hypothesized to involve direct viral invasion of skin cells, immune-complex mediated vasculitis, and microvascular thrombosis secondary to the procoagulant state induced by COVID-19 [6].

A surge of primary COVID-19 infections occurred in China between November 2022 and March 2023 after the COVID-19 lockdown policies were suspended. The surge was most likely due to two Omicron variants, BA.5 and BF.7 [7]. Our study was designed to analyze the cutaneous manifestations associated with primary Omicron infection during this special period.

## Participants and methods

### Ethics approval and consent to participate

This study was reviewed and approved by the Ethics Committee of Peking University Third Hospital (Beijing, China). The study was approved by the Medical Science Research Ethics Committee of Peking University Third Hospital (Approval No. IRB00006761-M2023102). The Ethics Committee granted a waiver of informed consent because this was a retrospective study analyzing de-identified clinical data, and the research posed no risk to the participants. All procedures were conducted in accordance with the Declaration of Helsinki.

### Study population and selection criteria

We conducted a retrospective analysis of participants diagnosed with the primary COVID-19 infection and cutaneous manifestations at our Out-Patient Unit in the Dermatology Department of Peking University Third Hospital between November 2022 and April 2023. The initial screening of the Hospital Information System (HIS) identified 103 participants. The inclusion criteria were: (1) age ranging 2–90 years, children under 2 years were excluded due to the significant differences in cutaneous immune responses and the difficulty in obtaining reliable symptom reports in this age group; (2) both males and females; (3) typical COVID-19 symptoms including fever, cough, headache, myalgia, arthralgia, and diarrhea; (4) the primary COVID-19 infection in naive individuals; (5) laboratory-confirmed COVID-19 positivity (based on colloidal gold immunochromatography, ELISA, or PCR); (6) unexplained skin rash within 4 weeks of COVID-19 symptom onset without otherwise apparent triggers. The exclusion criteria included: (1) negative or absent COVID-19 test results; (2) pre-existing severe systemic or psychiatric illnesses (including malignancies, severe cardiopulmonary failure, advanced hepatic or renal disease, and other infectious diseases); (3) prior COVID-19 infection, as repeated infections may result in pre-existing humoral and cellular immunity that significantly alters the host immune response, thereby confounding the characterization of cutaneous manifestations attributable to primary infection; (4) suspected drug eruptions, which were excluded based on detailed medication history review, clinical morphology assessment, and the temporal relationship between drug intake and rash onset, with further exclusion of cases where rash resolved upon drug discontinuation, and (5) incomplete medical records. The study protocol was reviewed and approved by the IRB at the Peking University Third Hospital. The study protocol was developed in accordance to the ethical principles set forth by the Declaration of Helsinki.

Wheel vasculitis was distinguished from wheels based on the following criteria: lesion duration exceeding 24 hours, residual purpura or hyperpigmentation after lesion resolution, and, where clinically indicated, histopathological examination confirming leukocytoclastic vasculitis. Cases with features suggestive of wheel vasculitis were excluded from the wheels category.

### Data collection

For each participant, we collected the following: demographics including sex/gender, age, and race, primary COVID-19 symptoms including fever, e.g., temperature and fever duration, respiratory symptoms, e.g., sore throat and cough, and peripheral neurological symptoms, e.g., myalgia and arthralgia. SARS-CoV-2 variant genotyping was performed for all participants using next-generation sequencing, and all cases were confirmed to be infected with the Omicron variant. Cutaneous manifestations were evaluated in detail, including time of onset relative to the systemic COVID-19 symptoms, morphology of the rash, distribution, and potential contributing factors. The treatment of cutaneous manifestations including traditional Chinese medications was retrieved from the HIS. Medical records and the HIS were accessed for research purposes between 01/04/2023 and 30/12/2023. During and after data collection, the authors did not have access to information that could identify individual participants.

### Statistical analysis

Data analysis was performed using IBM SPSS version 27.0. Categorical variables were expressed as frequencies and percentages, with inter-group comparisons conducted using chi-square tests or Fisher’s exact tests. The normality of continuous variables was assessed using the Shapiro-Wilk test. Normally distributed data were presented as mean and standard deviation, while non-normally distributed data was expressed as median and interquartile range (IQR). For normally distributed data meeting the assumption of homogeneity of variance, one-way analysis of variance was employed for multi-group comparisons. For data violating these assumptions, the Kruskal-Wallis test was utilized for overall group comparisons. Multinomial logistic regression analysis was performed to identify potential risk factors associated with different types of cutaneous manifestations, with rash type (wheels, edematous erythema, and papule/papulovesicular eruptions) as the dependent variable and papulovesicular eruptions as the reference category. Allergy history and medication type were included as independent variables. P < 0.05 was considered statistically significant.

## Results

### General characteristics and systemic COVID-19 symptoms

**Fig 1:**
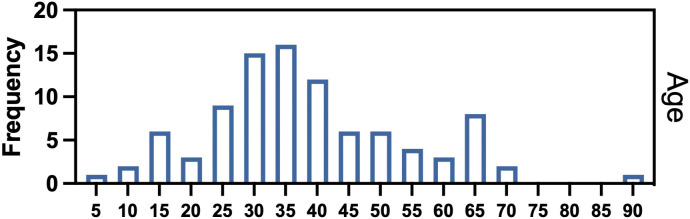
Demographic distribution of age.

A total of 94 participants with primary COVID-19 infection were included in the final analysis who requested dermatology counseling on cutaneous manifestations, representing 3.73% (94/2523) of all Omicron-variant COVID-19 patients presenting to our hospital during the same period. The general characteristics were summarized in Table 1. The age was ranging between 7 and 89 years old (mean ± SD 38.38 ± 15.88 years), with the majority (66.0%) was within 25–50 years old (Fig 1). A significant gender disparity was noted with a male-to-female ratio of 1:2.76 (p < 0.001). This phenomenon may be attributable to greater healthcare-seeking propensity in females, as well as the relatively more susceptible and dysregulated immune system in women, which renders them more prone to developing diverse cutaneous disorders.

**Table 1:** General characteristics and comparison among different types of cutaneous manifestations in patients with primary Omicron variant SARS-CoV-2 infection.

		Total (n = 94)	Wheels (n = 32)	Erythema (n = 49)	Papule/Papulovesicular Eruptions (n = 13)	*F/H/C*	*P*
Cutaneous Manifestations	Pruritus (VAS)	4 (2 7)	6 (4.25, 8)	3 (0.75, 5.5)	3 (0, 5)	13.677	0.001
	Pain (VAS)	0 (0, 1)	0 (0, 0)	0 (0, 3)	0 (0, 1.5)	10.713	0.005
Rash Distribution	Head and Face	11 (11.7)	0 (0)	6 (12.2)	5 (38.5)		0.009
	Trunk	5 (5.3)	2 (6.3)	2 (4.1)	1 (7.7)		
	Extremities	31 (33.0)	9 (28.1)	18 (36.7)	4 (30.8)		
	Generalized	47 (50.0)	21 (65.6)	23 (46.9)	3 (23.1)		
Age		36.0 (28.0, 48.0)	37.5 (31.3, 54.0)	34.0 (27.0, 48.0)	36.0 (27.0, 45.5)	2.606	0.272
Sex	Female	69 (73.4)	24 (75.0)	35 (71.4)	10 (76.9)	0.222	0.895
Peak Body Temperature		38.41 ± 0.62	38.34 ± 0.52	38.39 ± 0.63	38.68 ± 0.76	1.486	0.232
Duration of Fever		2 (1 3)	2 (2 3)	2 (1 3)	2 (1 3)	0.975	0.614
Fever-to-Rash Interval		3 (2 12)	3 (2.25, 7)	3 (2 14)	14 (2 21)	1.664	0.435
Systemic Symptoms	Cough	74 (78.7)	27 (84.4)	36 (73.5)	11 (84.6)		0.605
	Sore Throat	32 (34.0)	10 (31.3)	18 (36.7)	4 (30.8)		0.651
	Myalgia	48 (51.1)	17 (53.1)	23 (51.1)	8 (61.5)	0.441	0.802
	Arthralgia	6 (6.4)	1 (3.1)	5 (10.2)	0 (0.0)		0.246
History of Allergies		32 (34.0)	9 (28.1)	18 (36.7)	5 (38.5)		0.523
Medication Type	TCM	7 (7.4)	2 (6.3)	4 (9.1)	1 (7.7)		0.006
	Western Medicine	35 (37.2)	15 (46.9)	11 (25.0)	9 (69.2)		
	TCM+Western Medicine	19 (20.2)	2 (6.3)	16 (36.4)	1 (7.7)		
	No Medication	28 (29.8)	13 (40.6)	13 (29.5)	2 (15.4)		

The predominant clinical symptom of COVID-19 infection was fever, with a mean body temperature of 38.41 ± 0.62 °C. Fever duration averaged 2 days (range 1–3 days). Respiratory symptoms were primarily cough (78.7%) and sore throat (34.0%), while peripheral neurological symptoms included myalgia (51.1%) and arthralgia (6.4%). No severe pulmonary complications were observed for all participants.

### Cutaneous manifestations

**Fig 2:**
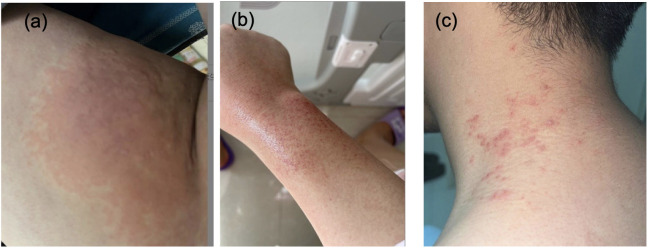
Representative clinical images of the three major types of cutaneous manifestations and four sub-types of erythema after COVID-19 infection. The three major types were wheals (a), erythema (b) and papule/papulovesicular eruptions (c), respectively.

The cutaneous manifestations associated with primary COVID-19 infection predominantly presented as three distinct types: wheals (n = 32, 34%), erythema (n = 49, 52%), and papule/papulovesicular eruptions (n = 13, 14%). The major lesions of wheels (Fig 2a) presented as transient wheals of varying sizes and durations, lasting from several hours to several days and predominantly widespread (66%). Of the 49 erythematous cases, none fulfilled the diagnostic criteria for erythroderma, which requires erythema involving ≥90% of the body surface area; the generalized erythema observed in this cohort refers to widespread erythematous lesions covering a significant but sub-erythrodermic body surface area. Drug eruptions were systematically excluded in all cases based on detailed medication history, clinical morphological assessment, temporal relationship between drug intake and rash onset, and clinical course. Erythema was classified into four subtypes: (1) diffuse edematous erythema (Fig 2b, 17/49, 34.7%) without well-defined borders, commonly affecting the bilateral eyelids, cheeks, and extensor surfaces of the limbs, occasionally accompanied with dense, fine, needle-like purpura on the proximal extremities; (2) atopic dermatitis-like lesions (S1a Fig, 15/49, 30.6%), characterized by dry erythema with fine scaling, predominantly on the upper eyelids, cubital fossae, and popliteal fossae; (3) pityriasis rosea-like erythema (S1b Fig, 11/49, 22.4%), primarily distributed on the trunk; (4) erythema multiforme-like lesions (S1c Fig, 6/49, 12.2%), mainly affecting the proximal extremities. Multiple subtypes coexisted only in one participant, who had diffuse edematous erythema on the extremities observed with other erythema-like lesions. Papule/papulovesicular eruptions (Fig 2c) were widely distributed across the trunk and extremities, demonstrating a predilection for the head and face (39%).

The median time from fever onset to the appearance of cutaneous manifestations was 3 days (IQR: 2–12 days). The temporal relationship between fever onset and rash varied among the different types (S2 Fig). Wheels and edematous erythema showed similar latency, with a median of 3 days. In contrast, papule/papulovesicular eruptions exhibited various latencies with a median onset of 14 days post-fever. While a 65-year-old male participant developed a rash on the day of fever, three female participants aged 27-, 36-, and 40-years experienced a rash on day 21, and a 36-year-old female developed a rash 28 days after the fever.

The skin lesions were widespread, involving the head, face, trunk, and extremities in 50% of participants. The remaining 50% exhibited localized symmetrical distributions, with 33% confined to the extremities and 12% limited to head and face. The distribution patterns of the rashes differed significantly among the three types (p = 0.009). Wheels was predominantly generalized (66%), with 28% confined to the extremities and none exclusively affecting the head and face. Edematous erythema showed a more varied distribution: 47% generalized, 37% limited to the extremities, and 12% confined to the head and face. Papule/papulovesicular eruptions were less frequently generalized (23%), with a predilection for the head and face (39%) and extremities (31%), and less commonly affecting the trunk (8%).

Pruritus and pain associated with the skin lesions were quantified using a Visual Analogue Scale (VAS). Pruritus was common with a median VAS of 4 (IQR 2–7). Although pain was much less common with a median VAS of 0 (IQR 0–1) and 67% of participants (n = 63) reported no pain (VAS 0), a significant pain (VAS ≥ 5) was noted among 6 participants. Wheel wheals were significantly associated with pruritus (median VAS 6) than that of edematous erythema and papule/papulovesicular eruptions (both median VAS 3, p = 0.001). No participants with wheels reported pain, 49% of those with erythema and 23% with papule/papulovesicular eruptions experienced varying degrees of pain (p = 0.005).

### Analysis of demographic and clinical correlates

No statistically significant association was noted between cutaneous manifestations, demographic characteristics or COVID-19 symptoms. The demographic characteristics included in the analysis were age (p = 0.272) and sex/gender (p = 0.895), and the COVID symptoms were fever intensity (p = 0.232), fever duration (p = 0.614), respiratory symptoms (cough p = 0.605, sore throat p = 0.651), and peripheral symptoms (myalgia p = 0.802, arthralgia p = 0.246).

Analysis of allergy history revealed a lower prevalence among participants with wheels (28%) compared to those with edematous erythema (37%) and papule/papulovesicular eruptions (39%), although this difference was not statistically significant (p = 0.523). Examination of medication use during COVID-19 infection indicated that participants with wheels were primarily treated with Western medicine (47%), followed by not treated with specific COVID-19 medications (41%) and traditional Chinese medicine alone or in combination with Western Medicine (6%). Participants with edematous erythema were treated with various medications, and those with papule/papulovesicular eruptions were treated predominantly with Western medicine (69%).

Multinomial logistic regression analysis was used to investigate the association of allergy history and medication type with rash occurrence. Assuming no interaction between allergy history and medication type (Table S1 in S1 File), neither allergy history (p = 0.563, OR=0.662, 95% CI: 0.164–2.679) nor medication type (TCM: p = 0.387, OR=0.286, 95% CI: 0.017–4.892; Western medicine: p = 0.127, OR=0.265, 95% CI: 0.048–1.462; Combined TCM and Western medicine: p = 0.407, OR=0.302, 95% CI: 0.018–5.106) showed significant associations with wheels or papule/papulovesicular eruptions. Similarly, no significant differences were observed for allergy history (p = 0.831, OR=1.162, 95% CI: 0.293–4.618) or medication type, although using Western medicines showed a borderline significance (p = 0.057, OR=0.186, 95% CI: 0.033–1.052) comparing edematous erythema and papule/papulovesicular eruptions.

Analyses of medical history revealed that 32% of participants had a history of allergies, including sensitivity to pollen, dust mites, fungi, and pet dander. During COVID-19 infection, 30% of participants had no pharmacological intervention. Among the participants who received the treatment, 35 (37%) were treated exclusively with Western medicines, primarily non-steroidal anti-inflammatory drugs (NSAIDs), e.g., ibuprofen, and antiviral agents such as nirmatrelvir and ritonavir. 7 participants (7%) were treated with traditional Chinese medicine (TCM) exclusively, with Lianhua Qingwen capsules, an antipyretic and detoxifying formulation, being the most used. Combinations of Western and TCM were used in 19 participants (20%). A potential interaction between allergy history and medication type (Table S2 in S1 File) was identified among participants with wheels *vs.* papule/papulovesicular (p = 0.06, OR=0.133). However, no significant interactions were observed in the edematous erythema *vs.* papule/papulovesicular.

## Discussion

The clinical presentations of COVID-19 have evolved alongside the emergence of successive variants, and available dermatological evidence remains limited. During the Alpha-predominant period, a Spanish study reported cutaneous manifestations in 47% of COVID-19 patients (n = 375), including maculopapular eruptions (47%) and wheels (19%), with acral lesions resembling chilblains also observed, particularly in younger individuals [8]. The Delta variant was associated with vasculitic rashes and erythema multiforme, alongside persistent maculopapular eruptions [9]. With the emergence of the Omicron variant, some studies have suggested a relatively attenuated pro-inflammatory profile, including diminished IFN responses and lower levels of IL-1β, IL-6, and TNFα [10]. However, the corresponding cutaneous manifestations of Omicron infection in the Chinese population have not been well characterized.

The skin lesions in our analysis presented a wide spectrum of clinical phenotypes, including all reported manifestations of earlier COVID-19 variants (Alpha and Delta). The increased incidence of cutaneous manifestations associated with the Omicron variant can be attributed to several factors. Firstly, the mutations in the Omicron spike protein have potentially altered its tissue tropism and interactions with host cells, including those in dermal and epidermal tissues [11]. This altered cellular affinity may contribute to the distinct patterns of skin lesions as identified. Secondly, the Omicron variant may elicit immune responses characterized by a diminished interferon response and an altered cytokine profile [12], resulting in a more balanced Th1/Th2 response, unlike the predominant Th1 responses from the earlier alpha and delta variants. Thus, these different immune responses might likely lead to different manifestations as well as the prevalence of cutaneous eruptions.

Analysis of the temporal relationship between fever onset and the appearance of cutaneous manifestations provided further insights into Omicron’s unique pathophysiology. Unlike other viral exanthems, such as measles (typically 4 days post-fever onset) and varicella-zoster virus infections (approximately 3 days), the timing of rash onset in COVID-19 infections showed considerable variability. Notably, a subset of patients, predominantly female, exhibited a prolonged interval between fever onset and rash appearance. This extended latency period may reflect Omicron’s distinctive pattern of immune dysregulation. The protracted but relatively mild inflammatory response associated with Omicron infections could lead to a gradual perturbation of immune homeostasis, particularly in individuals with certain predispositions. This phenomenon not only underscores the unique immunomodulatory properties of Omicron but also suggests potential gender-specific variations in immune responses to SARS-CoV-2. These observations collectively highlight the complex interplay between viral factors, host immune responses, and individual patient characteristics in shaping the clinical presentation of Omicron infections.

In our analysis, we carefully considered potential confounding factors that might influence the presentation and interpretation of cutaneous manifestations. These included patient demographics, allergy history, and medication use during COVID-19 infection. While our multinomial logistic regression analysis did not reveal statistically significant associations between these factors and specific rash types, the interaction between allergy history and Western medicine use approached significance in the comparison between wheels and papule/papulovesicular eruptions. This suggests a potential modulating effect of these factors on the cutaneous response to Omicron infection, albeit not reaching statistical significance in our cohort. The lack of strong associations with these potential confounders lends credence to the hypothesis that the observed skin manifestations are primarily driven by the Omicron variant’s unique pathophysiology and host immune interactions, rather than external factors. However, it is important to note that the relatively small sample size in some subgroups may have limited our ability to detect subtle influences. Future larger-scale studies may provide further insights into the role of these and other potential confounding factors in the dermatological manifestations of Omicron and other SARS-CoV-2 variants.

Future research integrating bioinformatics approaches holds considerable promise for advancing our understanding of the molecular mechanisms underlying Omicron-associated cutaneous manifestations. The detection of SARS-CoV-2 spike protein via immunohistochemistry (IHC) in pityriasis rosea-like and wheels-like skin lesions, as reported by Welsh et al., provides direct histopathological evidence of viral tropism for dermal endothelial cells and perivascular lymphocytes [6]. Building upon these insights, high-throughput transcriptomic and proteomic analyses of skin biopsies from Omicron-infected patients could further elucidate the specific host-pathogen interaction networks driving distinct cutaneous phenotypes. Specifically, single-cell RNA sequencing (scRNA-seq) of lesional skin would enable high-resolution characterization of infiltrating immune cell populations. This approach could uncover key cellular mediators and signaling pathways—such as type I interferon signaling, Th2 skewing, and mast cell activation—that differentiate wheel, erythematous, and papulovesicular eruptions at the molecular level. Furthermore, network-based bioinformatics and spatial transcriptomics could resolve the tissue-level distribution of viral particles and immune infiltrates within cutaneous lesions, complementing conventional IHC findings. Collectively, these bioinformatics-driven strategies will not only deepen mechanistic insights into the dermatological impact of Omicron but also provide a robust framework for developing biomarker-based diagnostic tools and targeted interventions.

### Limitations

Our analysis had limitations. Our participants were exclusively from the outpatient setting, suggesting a possible selection bias that the absence of inpatient data from severe COVID-19 cases precluded the conclusion whether severe cases are equally prone to similar cutaneous manifestations. Another notable limitation of this study is the sex imbalance in the study population, with females accounting for 73.4% of participants. This imbalance may limit the generalizability of findings to male patients and could potentially affect the regression results, as sex-related differences in immune response and drug metabolism may influence the type and severity of cutaneous manifestations. Future studies with more balanced sex distribution are warranted to validate these findings.

## Conclusion

This analysis has provided valuable insights into the cutaneous manifestations associated with the Omicron variant of COVID-19. Our findings have revealed dermatological presentations, predominantly characterized by wheels, erythema, and papule/papulovesicular eruptions. These cutaneous manifestations will not only expand our understanding of the clinical spectrum resulting from the Omicron variant but also highlight the evolving nature of COVID-19 and its dermatological impact.

## Supporting information

S1 Fig Erythema was then classified into four subtypes: the diffuse edematous erythema (Fig 2b) without clear border on bilateral eyelids, cheeks and extensor side of extremities, sometimes dense tiny needle-like purpura being found on proximal extremities; atopic dermatitis-like lesions (a), dry erythema with tiny scales on upper-eyelids, cubital fossa and popliteal fossa; pityriasis rosea-like erythema (b) mainly on trunk; erythema multiforme-like lesions (c) mainly on proximal extremities. (PNG)

S2 Fig Distribution of fever-to-rash intervals among different types of cutaneous manifestations. (PNG)

S1 File Multinomial logistic regression analysis of rash types in relation to allergy history and medication type. Table S1: analysis assuming no interaction; Table S2: analysis including interaction effects.
(DOCX)
