# Supplementary figures and images for: Skin manifestations of primary COVID-19 infection with the omicron variant
Source: PLoS One. 2026 Jul 17;21(7):e0352201. doi: 10.1371/journal.pone.0352201 (PMC13378995; doi:10.1371/journal.pone.0352201)

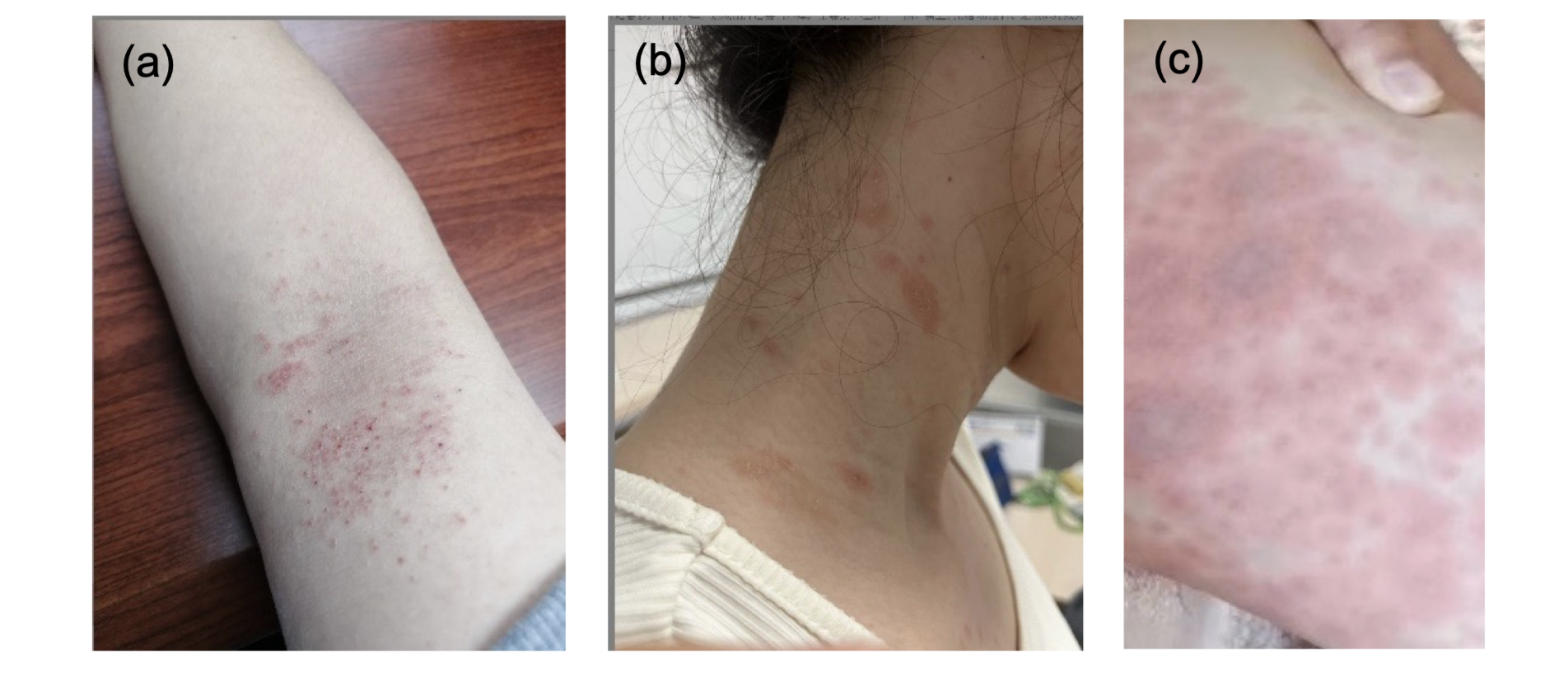

Supplement: S1 Fig — (PNG) [file pone.0352201.s001.png]

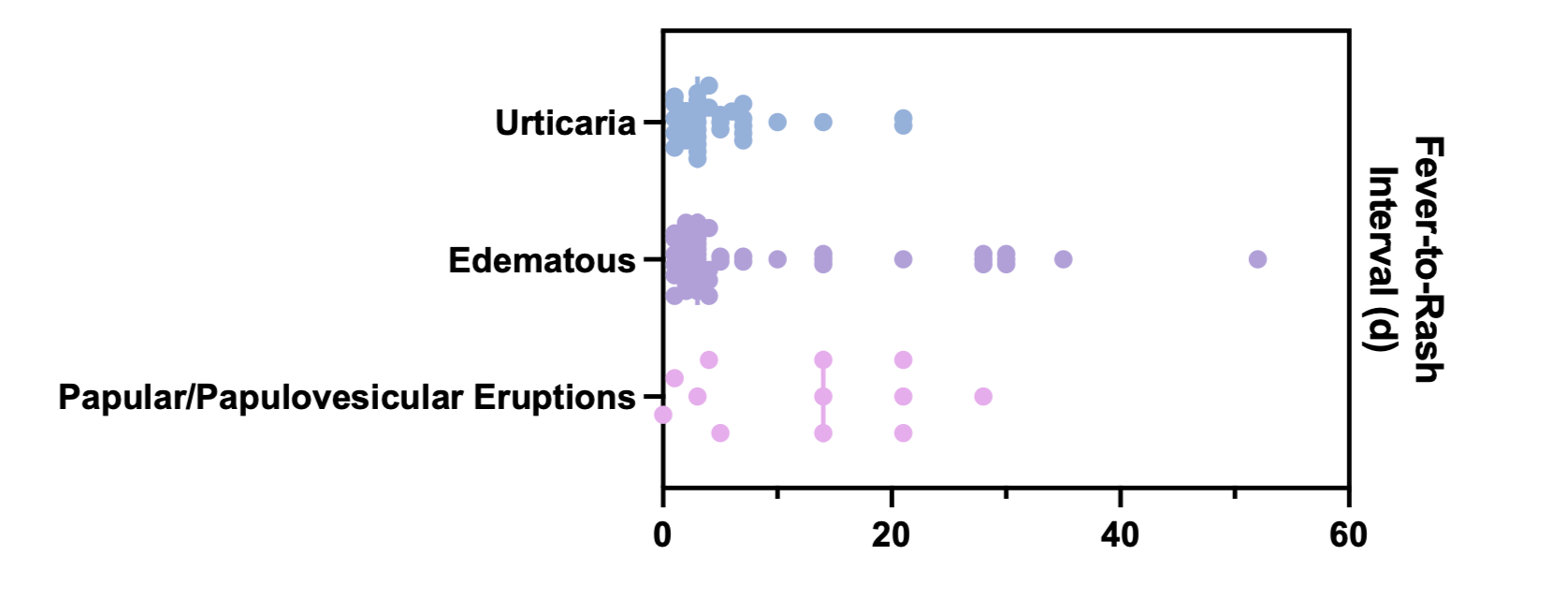

Supplement: S2 Fig — (PNG) [file pone.0352201.s002.png]
