# Supplementary material for: Skin manifestations of primary COVID-19 infection with the omicron variant
Source: PLoS One. 2026 Jul 17;21(7):e0352201. doi: 10.1371/journal.pone.0352201 (PMC13378995; doi:10.1371/journal.pone.0352201)
Supplement: S1 File — Table S1: analysis assuming no interaction; Table S2: analysis including interaction effects. (DOCX) [file pone.0352201.s003.docx]

**Table S1.** Multinomial logistic regression analysis of rash types in relation to allergy history and medication type (assuming no interaction)

| Variable Groups | Risk Factor | P | OR | 95% CI of OR | |
| --- | --- | --- | --- | --- | --- |
|  |  |  |  | Lower Limit | Upper Limit |
| Wheals/Papulovesicular Eruptions | Intercept | 0.012 |  |  |  |
|  | History of allergy/No history of allergy | 0.563 | 0.662 | 0.164 | 2.679 |
|  | TCM/No Medication | 0.387 | 0.286 | 0.017 | 4.892 |
|  | Western Medicine/No Medication | 0.127 | 0.265 | 0.048 | 1.462 |
|  | TCM+Western Medicine/No Medication | 0.407 | 0.302 | 0.018 | 5.106 |
| Edematous Erythema/Papulovesicular Eruptions | Intercept | 0.023 |  |  |  |
|  | History of allergy /No history of allergy | 0.831 | 1.162 | 0.293 | 4.618 |
|  | TCM/No Medication | 0.738 | 0.635 | 0.044 | 9.12 |
|  | Western Medicine/No Medication | 0.057 | 0.186 | 0.033 | 1.052 |
|  | TCM+Western Medicine/No Medication | 0.478 | 2.48 | 0.201 | 30.547 |

**Table S2.** Multinomial logistic regression analysis of rash types in relation to allergy history and medication type (including interaction effects)

| Variable Groups | Risk Factor | P | OR | 95% CI of OR | |
| --- | --- | --- | --- | --- | --- |
|  |  |  |  | Lower Limit | Upper Limit |
| Wheals/Papulovesicular Eruptions | Intercept | 0.054 |  |  |  |
|  | History of allergy * TCM |  | 0.547 | 0.547 | 0.547 |
|  | History of allergy * Western Medicine | 0.060 | 0.133 | 0.016 | 1.085 |
|  | History of allergy * TCM+Western Medicine | 0.998 | 13740241 |  |  |
|  | History of allergy * No Medication | 0.997 | 17990725 |  |  |
|  | No history of allergy * TCM | 0.577 | 0.444 | 0.026 | 7.666 |
|  | No history of allergy * Western Medicine | 0.677 | 0.667 | 0.099 | 4.478 |
|  | No history of allergy * TCM+Western Medicine | 0.995 | 2.90E-08 |  |  |
|  | No history of allergy * No Medication | Reference | | | |
| Edematous Erythema/Papulovesicular Eruptions | Intercept | 0.080 |  |  |  |
|  | History of allergy * TCM | 0.998 | 69616970 |  |  |
|  | History of allergy * Western Medicine | 0.227 | 0.300 | 0.043 | 2.112 |
|  | History of allergy * TCM+Western Medicine | 0.998 | 30915541 |  |  |
|  | History of allergy * No Medication | 0.997 | 25299457 |  |  |
|  | No history of allergy * TCM | 0.837 | 0.750 | 0.048 | 11.648 |
|  | No history of allergy * Western Medicine | 0.262 | 0.312 | 0.041 | 2.384 |
|  | No history of allergy * TCM+Western Medicine | 0.401 | 3.000 | 0.232 | 38.875 |
|  | No history of allergy * No Medication | Reference | | | |
